# *Cinnamomum burmannii* (Nees & T. Nees) Blume and *Eleutherine palmifolia* (L.) Merr. extract combination ameliorate lipid profile and heart oxidative stress in hyperlipidemic mice

**DOI:** 10.14202/vetworld.2020.1404-1409

**Published:** 2020-07-22

**Authors:** Retno Susilowati, Abdul Malik Setiawan

**Affiliations:** 1 Department of Biology, Faculty of Science and Technology, State Islamic University of Maulana Malik Ibrahim Malang, Malang 65144, East Java, Indonesia; 2 Department of Microbiology, Faculty of Medicine and Health Sciences, State Islamic University of Maulana Malik Ibrahim Malang, Malang 65144, East Java, Indonesia

**Keywords:** antihyperlipidemic, *Cinnamomum burmannii*, *Eleutherine palmifolia*, lipid profile, malondialdehyde, superoxide dismutase

## Abstract

**Background and Aim:**

Hyperlipidemia is an important risk factor for cardiovascular disease. The use of statins has adverse side effects that result in oxidative stress disorders. The objective of this study was to investigate the antihyperlipidemic effect of a combination of *Cinnamomum burmannii* and *Eleutherine palmifolia* extract in high-fat diet (HFD)-induced hyperlipidemia mice.

**Materials and Methods:**

Mice were divided into eight groups (n=4): Control group or healthy mice (normal), HFD-induced hyperlipidemic mice without any treatment (CE0), HFD-induced hyperlipidemic mice treated with 3.6 mg/kg body weight (BW) atorvastatin (atorvastatin), and HFD-induced hyperlipidemic mice treated with a combination of *C. burmannii* and *E. palmifolia* in the following ratios: 300:0 (C300), 225:75 (C225), 150:150 (CE150), 75:225 (E225), and 0:300 (E300). Mice were fed a HFD for 4 months to induce hyperlipidemia. Total cholesterol, cholesterol oxidase-peroxidase aminophenazone (CHOD-PAP), triglyceride-glycerine, and fat serum were analyzed with colorimetric method. The measurement of superoxide dismutase was done with the xanthine oxidase method and malondialdehyde measurement was done with the thiobarbituric acid method.

**Results:**

Results showed an increase in antihyperlipidemic characteristics as the concentration of *E. palmifolia* extract (p<0.05) increased. Duncan’s multiple range test also showed an increase in anti-stress oxidation as the concentration of *C. burmannii* extract (p<0.05) increased.

**Conclusion:**

The E225 group showed the most potential as a safe, antihyperlipidemic agent characterized by improvement in lipid profile and antioxidant balance.

## Introduction

Hyperlipidemia is a medical condition characterized by the elevation of any or all lipid profiles and/or lipoproteins in the blood and is also called hypercholesterolemia or hyperlipoproteinemia [1,2]. Although elevated low-density lipoprotein cholesterol (LDL-C) is thought to be the best indicator of atherosclerosis risk, hyperlipidemia can be determined by elevation of total cholesterol (TC) or triglyceride (TG) levels, or by low levels of high-density lipoprotein-C (HDL-C) [3]. Elevated LDL-C and TGs in hyperlipidemia correlate to cardiovascular disease (CVD), diabetes [4], and hypertension [5]. Moreover, ischemic heart patients have increased oxidative stress levels characterized by an elevation in malondialdehyde (MDA) levels [6]. Therefore, hyperlipidemic therapy is needed to decrease cases of diabetes mellitus, CVD, and hypertension [4]. Statins are currently used for treatment of hyperlipidemia, but they cause side effects and myopathy. Side effects of statins commonly relate to skeletal muscles and include muscle cramping, soreness, weakness, and fatigue [7]. Safe hyperlipidemic treatment is needed as an alternative to long-term and costly statin treatment.

Phenol is an organic compound with antioxidant and lipid reduction potential [8,9]. Cinnamon (*Cinnamomum burmannii* [Nees & T. Nees] Blume) is rich in phenols and flavonoids, including cinnamyl alcohol, coumarin, acid cinnamic, cinnamaldehyde, anthocyanin, and essential oils with a glucose constituent, protein, crude fat, pectin, etc. *C. burmannii* exhibits high biological activity, acting as an analgesic, antibacterial, antidiabetic, antifungal, antioxidant, antirheumatic, antithrombotic, and antitumor agent [10]. The addition of 15% cinnamon powder or 20 mg/body weight (BW) to the daily diet for 35 days may reduce TC, TGs, and LDL significantly, but not as effective as a daily dose of atorvastatin 0.2 mg/kg BW [11]. Cinnamaldehyde is the main compound in cinnamon bark [12-14]. Administration of 20 mg/kg cinnamaldehyde for 4 weeks reduced TG levels and increased HDL in spontaneous diabetic mice model of type 2 diabetes (C57blks/j db/db mice) [15]. Bawang dayak (*Eleutherine palmifolia* (L.) Merr) is a plant endemic to Borneo, Indonesia. *E. palmifolia* has been used empirically in therapy for several diseases, such as breast cancer, hypertension, diabetes, high cholesterol, ulcers, colorectal cancer, and strokes. *E. palmifolia* contains a high level of quercetin [14], which reduces the expression of the lipid, oleic acid, and sterol regulatory element-binding protein-1, and also inhibits the transcription of β-hydroxy β-methylglutaryl-coenzyme A (HMG-CoA), glycerol-3-phosphate acyltransferase, acetyl-CoA carboxylase, and fatty acid synthetase [16]. Long-term hyperlipidemia is characterized by oxidative stress imbalance and results in serious health disorders, such as atherosclerosis, stroke, and heart attack. The increase in MDA lipid peroxide inhibits the contractile function of the heart muscle which directly activates p38 mitogen-activated protein kinase and causes muscle cell length reduction and peak shortening [17]. At present, supporting scientific data are lacking for the use of this plant in hyperlipidemia prevention.

*C. burmannii* and *E. palmifolia* have different dominant active compounds and mechanisms against hyperlipidemia. *C. burmannii* bark is rich in cinnamaldehyde, whereas *E. palmifolia* is rich in quercetin. To maximize the antihyperlipidemic activity of both plants, we used a combination extract. Therefore, this study aimed to investigate the potential effect of *C. burmannii* and *E. palmifolia* combination extract on the regulation of lipid profiles and antioxidant activity by measuring MDA levels in the heart muscle tissue of high-fat diet (HFD)-induced hyperlipidemic mice.

## Materials and Methods

### Ethical approval

Animal maintenance and handling were in accordance with Principles of Laboratory Animal Care (NIH publication no. 85-23, revised 1985) [18]. The type of feed was standard chow diet and water *ad libitum*. Mice were obtained from Rattus Breeding Centre, Malang, Indonesia. All the protocols for animal study were approved by Ethics Commission of the Faculty of Science and Technology of UIN Maulana Malik Ibrahim Malang (no. 015/EC/KEP. FST/2018).

### Preparation of plant extract

Bark of *C. burmannii* and bulbs of *E. palmifolia* were obtained from UPT Materia Medica, Lahore street No. 87, Pesanggrahan, Batu, East Java, Indonesia. The plants were dried at 60°C, mashed, and filtered through an 80-mesh filter. A 500 g of plant powder was dissolved in 96% ethanol (1:3, w:v) for 72 h. The filtrate was then evaporated with a rotary evaporator to obtain the concentrated extract.

### Study period and location

This study was conducted from April 15, 2019 until October 31, 2019 in Animal Physiology Laboratorium, Plant Physiology Laboratorium and Animal Room, Department of Biology, Faculty of Science and Technology, State Islamic University of Maulana Malik Ibrahim Malang, Indonesia and Biomedical Laboratorium, Muhammadiyah University of Malang, Indonesia.

### Design of animal experiment

Thirty-two female Balb/c mice of the same age (8 weeks) and weight (25 g) were selected for this study. Mice were acclimated for 7 days at 25-27°C under 12 h light-dark cycles. Mice were fed a standard chow diet and provided *ad libitum* access to water. Balb/c mice were then divided into eight groups (four mice/group): Control normal diet mice (normal), HFD-induced hyperlipidemic mice without any treatment (CE0), HFD-induced hyperlipidemic mice treated with 3.6 mg/kg BW atorvastatin (atorvastatin), and HFD-induced hyperlipidemic mice treated with a *C. burmannii* and *E. palmifolia* combination extract in the following ratios, 300:0 (C300), 225:75 (C225), 150:150 (CE150), 75:225 (E225), and 0:300 (E300). Hyperlipidemia was induced by a HFD, cholesterol, and propylthiouracil (PTU) administration. For 120 days, mice of all HFD treatment groups had *ad libitum* access to HFD pellets containing quail egg yolk (10%) and bovine fat (10%). Seventeen and one-half mg of cholesterol in 0.3 ml of oil was administered through oral gavage. Mice had *ad libitum* access to water containing 0.01% PTU [19].

### Serum and heart tissue isolation

After 120 days of treatment with the experimental diet, animals were sacrificed. Blood and heart tissue were isolated immediately. Blood was collected from the ventricles of all mice after fasting for 8 h. The blood samples were centrifuged at 1500 rpm for 15 min. Serum was collected and stored in −20°C for further analysis. Heart tissue was isolated and washed with phosphate-buffered saline (PBS). A 0.1 g of heart organ was homogenized by adding 1 ml PBS, pH 7.0-7.4. Then, the homogenate was centrifuged at 3000 rpm, 4°C for 15 min. Lysate heart tissue was isolated and stored at −20°C for further superoxide dismutase (SOD) and MDA measurement (manual procedure of catalog No. E-BC-K020-M, E-BC-K025-S).

### Lipid, SOD, and MDA measurement

Levels of TC, TGs, and LDL in serum were measured using an enzymatic colorimetric method [20] with a reagent of TC assay kit (CHOD-phosphate oxidase peroxidase [PAP] method) and TG assay kit (single reagent, glycerine PAP method; Elabscience^®^, China). Sample absorbance was measured with an EZ Read 400 Microplate Reader. SOD level was measured with the xanthine oxidase method [21] using an SOD activity assay kit (hydroxylamine method; Elabscience^®^). MDA was measured with the thiobarbituric acid method (Elabscience^®^).

### Statistical analysis

All of the experimental data were represented as mean±standard deviation. Analysis of variance and Duncan’s multiple range test were performed using SPSS version 16.0 for Windows (SPSS, Inc., Chicago, IL, USA). p<0.05 was considered statistically significant.

## Results

### Lipid profile of mouse blood serum

To evaluate the antihyperlipidemic effects of *C. burmannii* and *E. palmifolia*, we analyzed the changes in serum lipid profiles of HFD-induced hyperlipidemic mice. The results showed that all lipid profiles (TC, TGs, and LDL-C) were higher in the HFD-treated group than in the normal group (Table-1). The concentration of TC significantly (p<0.05) decreased in hyperlipidemic mice treated with the single and a combination extract of *C. burmannii* and a combination of *C. burmannii* and *E. palmifolia*. The decrease in TC in the C300 and C225 groups was close to the atorvastatin group. The levels of TC in the CE150, E225, and E300 groups were not significantly different than the normal and atorvastatin groups (p>0.05). Furthermore, the single extract of *C. burmannii* and in combination with *E. palmifolia* reduced TG and LDL-C significantly (p<0.05) compared to the hyperlipidemic group. The TGs of the C300 and C225 groups were close to the TGs of the atorvastatin group, while the levels of TGs and LDL-C in the E225 and E300 groups were not significantly different from normal (p>0.05).

**Table-1:** Serum lipid profile, MDA, and SOD activity of heart muscle of HFD-induced mice.

Treatment	Variable				

	TC (mg/dl)	TG (mg/dl)	LDL (mg/dl)	SOD (U/ml)	MDA (nmol/ml)
Normal	153.32±15.80^a^	48.48±7.70^a^	138.80±13.28^a^	10.04±0.47^bc^	2.40±0.26^b^
Atorvastatin	177.93±60.75^ab^	90.08±4.71^b^	139.59±8.32^a^	17.3 ±2.32^f^	2.40±0.29^b^
CE0	326.95±61.83^c^	181.93±36.47^e^	227.08±35.19^c^	7.43±0.73^a^	3.17±0.25^c^
C300	243.75±28.46^b^	95.42±4.49^b^	255.21±43.57^c^	13.5±1.40^e^	1.77±0.56^ab^
C225	210.35±52.41^b^	83.21±5.54^b^	219.53±47.85^bc^	12.76±0.80^de^	2.03±0.24^ ab^
CE150	190.04±48.05^ab^	132.83±18.82^c^	239.06±8.93^c^	10.56±1.16^bc^	1.80±0.48^ ab^
E225	193.36±31.42^ab^	53.95±6.31^a^	170.31±20.85^a^	11.16±1.01^cd^	1.96±0.70^ab^
E300	219.34±55.99^ab^	45.29±4.84^a^	173.96±43.63^ab^	8.71±1.50^ab^	1.54±0.69^a^
Statistical test	ANOVA p<0.01 DMRT α=0.05	ANOVA p<0.01 DMRT α=0.05	ANOVA p<0.01 DMRT α=0.05	ANOVA p<0.01 DMRT α=0.05	ANOVA p<0.01 DMRT α=0.05

Normal (healthy mice), atorvastatin (3.6 mg/kg BW of atorvastatin), CE0 (HFD-induced hyperlipidemia mice without treatment), C300 (HFD-induced hyperlipidemia mice treated with 300 mg/kg *C. burmannii*), C225 (HFD-induced hyperlipidemia mice treated with 225 mg/kg *C. burmannii* + 75 mg/kg * E. palmifolia*), CE150 (HFD-induced hyperlipidemia mice treated with 150 mg/kg *C. burmannii* + 150 mg/kg *E. palmifolia*), E225 (HFD-induced hyperlipidemia mice treated with 75 mg/kg * C. burmannii* + 225 mg/kg * E. palmifolia*), and E300 (HFD-induced hyperlipidemia mice treated with 300 mg/kg * E. palmifolia*). TC=Total cholesterol, TG=Triglyceride, LDL=Low-density lipoprotein, SOD=Superoxide dismutase, MDA=Malondialdehyde. HFD=High-fat diet, BW=Body weight, *E. palmifolia=Eleutherine palmifolia,* *C. burmannii=Cinnamomum burmannii*

### Oxidative stress in heart muscle of mice

**Figure-1:**
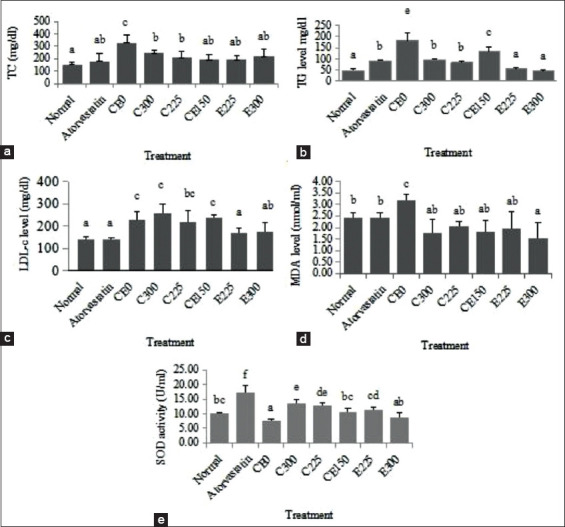
Lipid profile, malondialdehyde, and superoxide dismutase activity of heart muscle in high-fat diet-induced mice.

The level of oxidative stress was significantly increased in the HFD-treated group, which was characterized by an elevation in MDA levels and reduction in SOD activity. Interestingly, the single extract of *C. burmannii* and in combination with *E. palmifolia* significantly (p<0.05) reduced MDA levels compared to the hyperlipidemic group (CE0; Figure-1). All groups of combination extract (excluding E300) had significantly reduced MDA levels close to those of the atorvastatin group. Furthermore, the single and/or a combination extract of *C. burmannii* and *E. palmifolia* increased the activity of SOD significantly (excluding E300) compared to the hyperlipidemic group to normal level (p<0.05). The E300 group had a reduced MDA and increased SOD level close to that of the atorvastatin group (p>0.05).

## Discussion

The present study revealed that the combination of 150 mg/kg *C. burmannii* and 150 mg/kg *E. palmifolia* extract (CE150 group) and the combination of 75 mg/kg *E. palmifolia* and 225 mg/kg *C. burmannii* extract (E225 group) exhibited high reduction in lipid profile levels compared to the other treatment groups. This was supported by the antihyperlipidemic activity of each plant. Ethanolic extract of *C. burmannii* reduced lipid and lipoprotein profiles. Cinnamon consumption of 120 mg/day to 6 g/day for 4-18 weeks reduced TC, TGs, and LDL [22,23]. Patients with metabolic syndrome that consumes cinnamon (six capsules or three g) daily or wheat flour showed better results than Febrinda *et al*. [24] who reported ethanolic and water extract of *C. burmannii* in 100 mg/kg BW dose reduced TC and LDL in alloxan-induced diabetic mice (110 mg/kg BW) for 4 days before treatment. However, there was no significant effect on HDL and TG levels. The administration of cinnamon water extraction at a dose of 100 mg/kg BW for 6 weeks significantly reduced cholesterol and TGs in HFD- and alloxan-induced rat serum which previously increased HDL-C and reduced LDL-C serum [25]. Water-alcoholic extract of cinnamon at a dose of 100 mg/kg BW for 12 weeks reduced TC and TG blood serum levels, but they did not reach a normal level and lowered HDL to the same level as in a normal diet [26].

*C. burmannii* extract can act as an antihyperlipidemic agent due to the high amount of cinnamaldehyde in the bark. The level of cinnamaldehyde in ­cinnamon bark is about 65-80%, while in leaves is only 1-5%. In addition, cinnamon also contains eugenol, which is about 5-10% in the bark and about 70-95% in the leaves [27]. This hypothesis is supported by previous research which found that the addition of 2% cinnamaldehyde to the diet for 5 weeks significantly reduced the BW of obesity-induced mice. This finding also showed a reduction of TC and TGs resulting from cinnamaldehyde compound [28].

The antihyperlipidemic effects of combination extract are supported by the role of single plant extract of *E. palmifolia*, which has several active compounds in the bulb. The main bioactive compounds of the ethanolic extract of *E. palmifolia* are gallic acid (97.37 mg), epicatechin gallate (80.17 mg), quercetin (64.8 mg), *Eleutherine* (33.42 mg), and rutin (29.94 mg) [29]. This study showed better results than the previous one, which reported that 2% methanolic extraction of *E. palmifolia* for 1 week did not reduce TC and TGs in blood serum [30]. Daily administration of gallic acid protected against hepatic steatosis, obesity, hypercholesterolemia, and insulin resistance among HFD-induced nonalcoholic fatty liver disease mice [31]. Ethanol extract of *E. palmifolia* at a dose of 100-400 mg/kg BW for 14 days did not reduce the MDA level in an alloxan-induced diabeticrat [32]. This study demonstrated that combination extract could significantly increase SOD activity in heart muscle of hyperlipidemic mice. This finding was in line with other research which proved that ethanolic extract of *Cinnamomum zeylanicum* and *Cinnamomum cassia* at a dose of 600 mg/kg BW significantly increased the levels of SOD, glutathione peroxidase (GPx), and GSH in the liver of streptozotocin-induced rat [33]. The result is also supported by Yuanita *et al*. [30], who found that methanol extract of *E. palmifolia* significantly increased SOD and reduced MDA in chicken blood. The highest antioxidant activity of *E. palmifolia* was found through a DPPH test of extract obtained by 50% methanol [34], and a dose of 100-200 mg/kg BW increased the activity of SOD, GPx, and catalase but not to normal levels [25].

This study showed that the E225 group exhibited the best results for improving all parameters. This is in accordance with Prakash *et al*. [35], who proved that the four extracts together were more effective than a single extract or combination of two extracts against free radicals. Even though the components of the extract are the same, different amounts of each extract produce different results in reducing the levels of lipids, lipoproteins, and oxidative stress [36]. This might be caused by several different pathways for each parameter and different active compounds in the extract, resulting in different synergistic mechanisms.

Several studies have supported the antihyperlipidemic mechanisms of combination extract. Quercetin from *C. burmannii* can inhibit cholesterol synthesis by inhibiting the activity of HMG-CoA reductase [37]. Moreover, tannic acid and gallic acid have a high affinity for cholesterol [38]. In addition, the inhibitory effects on digestion and absorption of cholesterol in the gastrointestinal tract were induced by catechin, epicatechin, and epicatechin gallate from *E. palmifolia* [39]. The inhibition of lipids and cholesterol absorption during food digestion is very effective in ameliorating the increase of lipids and cholesterol through external channels, thereby reducing its accumulation in the body. Thus, *E. palmifolia* has more potential as an antihyperlipidemic than *C. burmannii*. Aside from being involved in HMG-CoA reductase inhibition, quercetin and kaempferol from *C. burmannii* also act as powerful natural antioxidants [40]. Hydrogen donation is the main mechanism of phenolics as antioxidants. The lower strength of the O–H bond present in phenolics corresponds to a higher scavenging activity. Quercetin and kaempferol identified in *Cinnamomum* species have a C2-C3 double bond and a C-3 hydroxyl group, while the most active hydroxyl groups are those attached to the C4 and C3 positions [41]. This research found that a daily dose composed of 225 mg/kg *E. palmifolia* and 75 mg/kg *C. burmannii* showed the most potential effect as an antihyperlipidemic agent.

## Conclusion

The combination of *C. burmannii* and *E. palmifolia* extract has more potential effect as a safe antihyperlipidemic agent, compared to single plant extracts, in improving blood lipid, and lipoprotein profiles and also preventing oxidative stress in the hearts of hyperlipidemic mice. The combination E225 extract (225 mg/kg *E. palmifolia* and 75 mg/kg *C. burmannii*) is a safe antihyperlipidemic agent due to its neutralization of oxidative stress, and normalization of lipid and lipoprotein levels, in hyperlipidemic mice.

## Authors’ Contributions

RS contributed to conceptual design, conducted the experiment and wrote the manuscript. AMS performed data analysis and revised the manuscript. Both authors read and approved the final manuscript.
